# Contralateral breast dose from partial breast brachytherapy

**DOI:** 10.1120/jacmp.v16i6.5296

**Published:** 2015-11-08

**Authors:** R. Cole Robinson, Christopher L. Nelson, Elizabeth S. Bloom, Kelly D. Kisling, Bryan E. Mason, Gary D. Fisher, Steven M. Kirsner

**Affiliations:** ^1^ Department of Radiation Physics The University of Texas M.D. Anderson Cancer Center Houston TX; ^2^ Department of Radiation Oncology The University of Texas M.D. Anderson Cancer Center Houston TX USA

**Keywords:** brachytherapy, breast cancer, partial breast, contralateral breast, SAVI

## Abstract

The purpose of this study was to determine the dose to the contralateral breast during accelerated partial breast irradiation (APBI) and to compare it to external beam‐published values. Thermoluminescent dosimeter (TLD) packets were used to measure the dose to the most medial aspect of the contralateral breast during APBI simulation, daily quality assurance (QA), and treatment. All patients in this study were treated with a single‐entry, multicatheter device for 10 fractions to a total dose of 34 Gy. A mark was placed on the patient's skin on the medial aspect of the opposite breast. Three TLD packets were taped to this mark during the pretreatment simulation. Simulations consisted of an AP and Lateral scout and a limited axial scan encompassing the lumpectomy cavity (miniscan), if rotation was a concern. After the simulation the TLD packets were removed and the patients were moved to the high‐dose‐rate (HDR) vault where three new TLD packets were taped onto the patients at the skin mark. Treatment was administered with a Nucletron HDR afterloader using Iridium‐192 as the treatment source. Post‐treatment, TLDs were read (along with the simulation and QA TLD and a set of standards exposed to a known dose of 6 MV photons). Measurements indicate an average total dose to the contralateral breast of 70 cGy for outer quadrant implants and 181 cGy for inner quadrant implants. Compared to external beam breast tangents, these results point to less dose being delivered to the contralateral breast when using APBI.

PACS number: 87.55.D‐

## I. INTRODUCTION

In 2011 over 230,000 women in the United States were newly diagnosed with invasive breast cancer.^(1)^ Many of these women will undergo lumpectomy, followed by radiation therapy. While the necessity of radiotherapy has been debated in the past, multiple studies have demonstrated a significant reduction of in‐breast tumor recurrence with adjuvant whole‐breast radiation.^2^, ^3^ For those patients who opt for radiation therapy a typical treatment regimen involves daily treatment five days per week for five to six weeks. Patients who have to travel some distance for treatment or for those with full time commitments, this can be an onerous task. One possible solution to this hardship is the use of accelerated partial breast irradiation (APBI). APBI involves the treatment of the at‐risk tissue surrounding the lumpectomy cavity, rather than the whole breast, using a 10 fraction, twice daily treatment protocol.^(4)^ This can be delivered either with external‐beam radiation therapy or high‐dose‐rate (HDR) brachytherapy. This allows the patient to complete her course of treatment in a single week, as opposed to the five or six weeks required by whole‐breast external beam therapy. In 2002, the FDA approved the use of the MammoSite single‐entry balloon (Hologic, Bedford, MA) which greatly increased the interest in brachytherapy‐based APBI. The original MammoSite protocol used a single catheter with a single central dwell point to treat a 1 cm rind of tissue surrounding the lumpectomy site.^(5)^ A phase III clinical trial was initiated to study the efficacy of APBI as compared to conventional whole‐breast irradiation.^(6)^ As brachytherapy APBI has evolved, more types of single‐entry implant devices have been introduced, utilizing multiple catheters. These new devices use the treatment planning computer to optimize dwell times and positions to deliver the best dose coverage to the target volume while minimizing the dose to adjacent critical structures, such as the skin and the chest wall. One of the concerns over this form of treatment has been the dose to the contralateral breast both from the treatment and the associated CT scans needed for the twice daily quality assurance (QA) of the device placement. Radiation treatments for breast cancer have been shown to increase the incidence of a second breast cancer in the contralateral breast.^(7)^ In this study, the dose to the contralateral breast for patients implanted with the Strut Adjustable Volume Implant (SAVI) device (Cianna Medical, Aliso Viejo, CA) was investigated. This measured dose was then compared to published values obtained for whole‐breast external beam contralateral breast dose. The relative contributions from the treatment delivery and the CT implant verification were measured and discussed. Measurements in this study were achieved using thermoluminescent dosimetry packets placed on the patient, as opposed to phantom measurements. This was done to retain the variability of patient size and geometry which was an important part of this study. This study will provide clinicians with the ability to determine the contralateral breast dose for their given brachytherapy APBI treatment and verification protocol.

## II. MATERIALS AND METHODS

In this study, the dose to the contralateral breast from APBI brachytherapy for 12 randomly selected patients treated at the University of Texas MD Anderson Cancer Center was measured. All patients were treated to a total dose of 34 Gy in 10 fractions over five treatments days (BID), using a SAVI single‐entry multicatheter applicator. Additionally, all patients were enrolled in the institution's clinical protocol studying the acute and late toxicity of APBI, which had been approved by the institutional review board. The patient flow for our APBI patients was first to visit our department for a cavity evaluation, then to proceed to patient simulation and planning, followed by the HDR treatments which include pretreatment verification scans. For the cavity evaluation and all subsequent CT scans, the patient was placed on a Philips Brilliance Big Bore CT scanner (Philips Healthcare, Andover, MA) for an AP and lateral scout view (120 kV, 50 mA). This was followed by a limited axial scan (miniscan) (120 kV, 250 mAs) encompassing the lumpectomy cavity.

A planning CT in the radiation oncology department was performed within 48 hrs of device placement by the surgeon in the operating room. The patient was placed supine on the CT couch and a limited CT scan was obtained through the device. The miniscan was evaluated by the radiation oncologist to confirm conformance of the struts of the SAVI to the edges of the lumpectomy cavity. For our criteria, the volume of seroma or air adjacent to the cavity (which could push target tissue out of the treatment volume) divided by the target volume to be treated (1 cm rind surrounding the cavity) must have been less than 10% of the targeted volume, which is in conformance with the NSABP protocol.^(6)^ Once device conformance was confirmed, a custom‐formed cradle was manufactured to reproduce daily patient positioning. AP and lateral scout views, followed by a full planning CT scan, was acquired including the whole breast with 2 cm margins both inferiorly and superiorly using 1.5 mm slices. The scout views were reviewed to determine the maximum strut width in both the AP and lateral view.

These widths were compared with subsequent pretreatment verification scouts to ensure the SAVI was expanded to the same extent with each treatment (within 2 mm). Patients were then given skin marks corresponding to SAVI strut location. These marks allowed for a daily check of applicator rotation. The CT images were transferred to a Nucletron Oncentra (Elekta Corp., Stockholm, Sweden) planning system. The patient was planned using methodology outlined in the literature.^8^, ^9^, ^10^ The patient was now ready to begin treatment.

Prior to each treatment, the patient was brought into the CT simulation suite to perform device positioning quality assurance. A typical treatment verification would commence with the patient being placed on the CT simulator couch in their custom cradle and aligned with skin laser marks. Subsequently, AP and lateral scout views were obtained. These scans were reviewed by the radiation oncologist and the physicist to check for rotation, correct expansion of the SAVI, and correct placement. If there were any concerns, a miniscan would be obtained through the device to confirm the device was in the same position and conformed to the lumpectomy cavity as originally planned. Once CT QA was completed, the patient was moved from the simulator to the HDR vault. The treatment device used was a Nucletron V3 HDR afterloader using Iridium‐192.

Contralateral breast dose measurements for this study were made with packets of TLD‐100 (Quantaflux, Dayton, OH) containing approximately 30 mg of LiF powder and measuring approximately 1 cm by 1 cm by 0.1 cm. Patients in this study had the contralateral breast dose measured for 2 or 3 of the 10 fractions treated, with the CT QA verification and the radiation HDR treatment measured separately. The TLD were placed at a measurement point on the patient's skin at the most medial aspect of the contralateral breast tissue (3 o'clock right breast, 9 o'clock left breast) as determined by the radiation oncologist. An ink mark was placed at this point to ensure continuity of subsequent measurements. Three TLD packets were taped to the patient's skin for each measurement clustered around this point. For CT QA TLD measurements, the packets would be placed on the patient for the AP and lateral scout scans and exposed during these scans. If it was decided to include a miniscan of the lumpectomy cavity at that time, the TLD packets were left in place and the miniscan was performed. As a result, the dose to the contralateral breast from the orthogonal scouts was included in any miniscan CT dose that was measured. After the QA was finished, the exposed TLD packets were set aside and the patient was escorted to the HDR treatment vault. The patient was positioned on the treatment table and three new TLD packets were taped to the measurement point. After treatment these packets would be removed from the patient and set aside for reading.

The last step of the measurement process was to expose three TLD to a known dose to be used as standards in the reading process. This was done on the same day as the patient TLD exposure. For this study, 100 cGy from 6 MV X‐ray Varian (Varian Medical Systems Inc., Palo Alto, CA) TG‐51–calibrated accelerator was used as a standard. The TLD were set on a large plastic water block, covered with 1.5 cm of bolus at an SSD of 100 cm (to the top of the bolus), 10×10 cm field size, and exposed to 100 MU.

The TLD were read by the Department of Radiation Physics at the University of Texas M.D. Anderson Cancer Center. TLD batch characteristics (e.g., linearity, fading) were measured with a Cobalt‐60 source that was traceable to the National Institute of Standards and Technology. The TLD used has an uncertainty of 5% in the range of 150 to 300 cGy.^(11)^ This uncertainty may increase with lower doses. The output of the reading process was corrected for energy response using energy correction factors. For the Ir‐192–exposed TLD, there is reasonably good agreement in the literature.^5^, ^6^, ^7^ An energy correction factor of 0.962 was used based upon the work of Davis et al.^(12)^ Energy correction factors for CT energy values however are more difficult to come by. A value of 0.952 was taken from Davis using a mean energy of 56 KeV.

## III. RESULTS

During the course of a patient's treatment, she typically underwent an average of four axial CT scans. The first scan occurred at the cavity evaluation where the radiation oncologist determined which size SAVI device and direction to implant. The second axial scan was an initial miniscan on planning day to determine extent of nonconformance and suitability of device placement to proceed with treatment planning. The third axial scan was the actual planning scan. For this study, the longer planning scan was assumed to contribute the same dose as a miniscan. An additional miniscan was usually performed during the course of treatment to verify correct positioning.

Table 1 shows the average dose to the contralateral breast as measured by TLD from the orthogonal scout views, miniscan, and HDR treatment per fraction, along with the standard deviation (SD) and range. Five of the patients in this study had TLD used to measure one or more of their miniscans and ten patients had TLD used to measure one or more of their scout scans (without miniscan).

Using the above data, a total estimated dose to the contralateral breast was calculated. For this study, the average patient was assumed to have been treated for 10 fractions, received nine scout scans (dose from one of the scout scans is included in a miniscan fraction) and four miniscans. This treatment regimen results in a dose of 116±73 cGy ((10×10.41)+(4×2.58)+(9×0.2)=116 cGy)) to the most medial point of the contralateral breast for the entire course of treatment, based on TLD measurements. Using the data in this table, it would be easy to estimate the contralateral breast dose for institutions using different APBI treatment regimens (i.e., a different number of scouts or miniscans).

Table 2 looks at total dose as a function of implant site. Five of the implants were recorded as inner quadrant, five as outer quadrant, and two were recorded as central implants. As would be expected, the inner quadrant implants result in more doses to the contralateral breast than the outer quadrant implants. The high standard deviations in Table 2 may be attributed to differences in exact implant site, patient geometry, which size SAVI was implanted, and the depth of the implant.

**Table 1:** Dose by source (cGy) per fraction

*Scan*	*No.*	*Avg.*	*SD*	*Range*
Miniscan	5	2.58	1.35	0.26–3.78
Scouts	10	0.2	0.37	0–1.61
Treatment	12	10.41	7.45	1.99–25.07

**Table 2:** Total contralateral breast dose by implant quadrant (cGy)

*Total Dose*	*No.*	*Avg.*	*SD*	*Range*
All	12	116	73	31–234
Inner quadrants	5	181	64	90–234
Outer quadrants	5	70	28	31–108

## IV. DISCUSSION

The data from this study indicates that APBI brachytherapy with the SAVI device results in low doses to the contralateral breast. The average dose of 116 cGy is a worst‐case result, given that the measurements were taken at the most medial point on the contralateral breast. An important outcome of this study is to compare the measured contralateral breast dose from SAVI brachytherapy treatment to measured contralateral breast doses from external beam therapy reported in the literature. On review, there is a large range of reported doses to the contralateral breast for a wide range of clinical whole‐breast radiotherapy techniques. Many studies report only the median dose to the contralateral breast, while other studies published data throughout the contralateral breast. As this research seeks to determine the worst‐case dose to contralateral breast tissue, data were pulled from studies listed in Table 3 using the high of the range data for inner quadrant measurements. Reported doses in Table 3 were for the entire course of treatment.

While a wide range of doses are reported, it should be noted that the lowest dose in Table 3 indicates a dose substantially above the study average of 116 cGy. Even in the case of inner quadrant implants, the study data still appear favorable to all but a few of the results listed in Table 3. If median dose to contralateral breast were the determinate dose to be considered for the contralateral breast, it is likely that the APBI brachytherapy advantage would be more substantial, given the point source nature of this form of treatment.

An additional concern with APBI brachytherapy has been the need for twice‐daily CT imaging for QA purposes and the resulting dose to the contralateral breast. Table 1 shows that minimal dose is delivered to the contralateral breast from these scans. An average number of scout images (9) per course of treatment would contribute approximately 3 cGy to the overall average of 116 cGy total dose. An average number of miniscans (4) would contribute approximately 10 cGy to the total dose. Thus, for the average patient, the twice‐daily CT scans contribute about 10% of the total dose to the patient's contralateral breast. It is worth noting that CT doses from external beam planning are not considered in the doses reported in Table 3. A typical external beam breast patient may undergo two to three CT planning scans, depending upon treatment technique (primary planning, boost, breath hold).

Future research in this area could include evaluating a median dose to the contralateral breast from APBI brachytherapy and looking at the contralateral breast dose from other single entry brachytherapy devices. Additionally, measuring the dose to the contralateral breast with evaluation of more variables such as SAVI size, implant distance from midline, and implant depth, could prove useful in guiding clinicians with their treatment decisions.

**Table 3:** External beam dose to contralateral breast (most medial)

*Study*	*Technique*	*Contralateral Breast Dose (cGy)*
Borghero^(11)^	Field in Field	226
Borghero^(11)^	Lateral Wedge	260
Borghero^(11)^	Medial and Lateral Wedge	299
Stovall^(7)^	Medial and Lateral Wedge	250
Stovall^(7)^	No Wedges	160
Zurl^(13)^	Free Breathing	150
Zurl^(13)^	Breath Hold	150
Fraass^(14)^	Tangent Fields	500
Kelly^(15)^	Half Beam + Wedge	390
Kelly^(15)^	Half Beam	345
Kelly^(15)^	Isocentric Tangents + Wedge	225
Kelly^(15)^	Asym Jaw + Block + Wedge	210
Kelly^(15)^	Isocentric Tangents	200
Kelly^(15)^	Asym Jaw + Block	190
Kelly^(15)^	Asym Jaw + Wedge	188
Kelly^(15)^	Asym Jaw	175

## V. CONCLUSIONS

The data from this study indicates that APBI brachytherapy with the SAVI interstitial applicator results in a lower dose to the contralateral breast, as compared to external beam whole‐breast irradiation. Additionally, twice‐daily CT scans for quality assurance account for approximately 10% of the total dose to the contralateral. Both of these findings help to support the use of single‐entry multicatheter device‐based partial breast irradiation as an alternative to external beam whole‐breast irradiation for patients who qualify.
